# Impacts of Multidisciplinary Lung Cancer Meeting Presentation in a Clinical Quality Registry

**DOI:** 10.1016/j.jtocrr.2026.100984

**Published:** 2026-03-06

**Authors:** Rob G. Stirling, Sanuki Tissera, Jessie Zeng, Mike Lloyd, Krupa Krishnaprasad, Lisa Briggs, Jacqueline Lesage, Tom Wood, Craig Underhill, Sagun Parakh, Louis Irving, Wasek Faisal, Robert Blum, Gary Richardson, Phillip Parente, Michelle Caldecott, Inger Olesen, Javier Torres, Evangeline Samuel, Christopher Lyne, Katharine See, David Langton, Tom John, Gavin M. Wright, Matthew Conron, James Bartlett, Nicola Atkin, Maggie Moore, Golsa Adabi, Luke Clegg, Nikolajs Zeps, Susan Harden, Zoe McQuilten, John Zalcberg

**Affiliations:** a School of Translational Medicine, Monash University, Melbourne, Victoria, Australia; b Department of Respiratory Medicine, Alfred Health, Melbourne, Victoria, Australia; c School of Public Health and Preventive Medicine, Monash University, Victoria, Australia; d Lung Cancer Advocate, Melbourne, Victoria, Australia; e University of New South Wales (NSW) Rural Medical School Albury, New South Wales, Australia; f Border Medical Oncology Research Unit, Albury Wodonga Regional Cancer Centre, Albury, New South Wales, Australia; g Olivia Newton-John Cancer Research Institute, Austin Hospital, Heidelberg, Victoria, Australia; h School of Cancer Medicine, La Trobe University, Bundoora, Victoria, Australia; i Department of Respiratory and Sleep Medicine, Royal Melbourne Hospital, Melbourne, Victoria, Australia; j Faculty of Medicine, Dentistry, and Health Sciences, University of Melbourne, Melbourne, Australia; k Department of Medical Oncology, Grampians Health, Ballarat, Victoria, Australia; l Department of Medicine, La Trobe University, Melbourne, Victoria, Victoria; m Bendigo Health Oncology Unit, Bendigo Health, Victoria, Australia; n Department of Medical Oncology, Cabrini Health, Malvern, Victoria, Australia; o Medical Oncology, Eastern Health, Box Hill, Victoria, Australia; p Department of Medicine, Epworth Healthcare, Richmond, Victoria, Australia; q Andrew Love Cancer Centre, Barwon Health, Geelong, Victoria, Australia; r Medical Oncology, Goulbourn Valley Health, Shepparton, Victoria, Australia; s Department of Medical Oncology, Latrobe Regional Health, Victoria, Australia; t Department of Respiratory Medicine, Monash Medical Centre, Clayton, Victoria, Australia; u Department of Respiratory Medicine, Northern Health, Epping, Victoria, Australia; v Department of Respiratory Medicine, Peninsula Health, Frankston, Victoria, Australia; w Department of Medical Oncology, Victorian Comprehensive Cancer Centre, Melbourne, Victoria, Australia; x Department of Cardiothoracic Surgery, University of Melbourne, Melbourne, Victoria, Australia; y Department of Surgery, St Vincent's Hospital, Melbourne, Victoria, Australia; z Department of Respiratory Medicine, St Vincent’s Hospital, Melbourne, Victoria, Australia; aa Department of Respiratory Medicine, Western Health, Footscray, Victoria, Australia; bb Parkville Integrated Palliative Care Service, Peter MacCallum Cancer Centre, Melbourne, Victoria, Australia; cc Department of Medical Oncology, Alfred Health, Melbourne, Victoria, Australia; dd Department of Radiation Oncology, Peter MacCallum Cancer Centre, Melbourne, Victoria, Australia; ee Sir Peter MacCallum Department of Oncology, University of Melbourne, Melbourne, Victoria, Australia

**Keywords:** Lung cancer, Multidisciplinary Meeting, Clinical quality registry, Quality Improvement, Benchmark reporting

## Abstract

**Background:**

Lung cancer is a heterogeneous and complex disease requiring multidisciplinary input for optimal management planning, with guidelines recommending that all patients be discussed in a multidisciplinary setting. Multidisciplinary meeting (MDM) discussion aims to enhance evidence-based management, improve treatment access, and optimize complex management plans.

**Methods:**

We aimed to assess the extent and impacts of MDM discussion in patients with lung cancer described by the Victorian Lung Cancer Registry from 2011 to 2023 in Victoria, Australia. We identified MDM-presented and nonpresented patients and assessed the impacts of MDM presentation. OR and survival hazard ratios were assessed using Cox proportional regression analysis. Survival analysis was determined using the Kaplan–Meier product-limit method. Sensitivity analyses were conducted using landmark analysis and propensity score matching methods.

**Results:**

A total of 18,597 patients were included, of whom 67% had evidence of presentation to a lung cancer MDM, with MDM presentation increasing from 59.1% to 80.6% during the study period. MDM presentation was associated with higher levels of provision of guideline-concordant treatment in NSCLC (56.2% versus 44.5%, *p* < 0.001), and lower levels of no treatment (10.0% versus 21.4%, *p* < 0.001). Modifiable factors that could increase MDM presentation include referral of patients of older age, stage IV disease, SCLC, and diagnosis at a private or regional hospital. Propensity-matched survival analysis in NSCLC revealed a median survival of 1.1 years for MDM-presented versus 0.86 years for nonpresented individuals, providing a 12% reduction in mortality hazard (hazard ratio 0.88 [0.82–0.95], *p* < 0.001).

**Conclusion:**

During the period of activity of the Victorian Lung Cancer Registry, MDM presentation increased from 59.1% to 80.6%. Management outcomes in MDM-presented patients identified multiple underserved cohorts and revealed considerable increases in treatment modalities and guideline-concordant treatment in NSCLC in this observational study, with an associated 12% improvement in survival advantage overall.

## Introduction

Lung cancer is a heterogeneous and complex disease that requires multidisciplinary input for optimal management planning, with national and international guidelines recommending that all patients with lung cancer be discussed in a multidisciplinary setting.^1^, ^2^, ^3^ The use of multidisciplinary meetings (MDM), or tumor boards, has become standard practice in many health systems to facilitate evidence-based decision making and optimized care. Despite these recommendations, there is substantial variability among institutions in the proportion of patients discussed at MDMs, perhaps driven by resourcing, institutional practice, geography, or staff availability, with reported rates ranging from 29.7% to 94%.^4^^,^^5^

Multidisciplinary discussion aims to improve access to treatment, increase the utilization of evidence-based management, and enhance coordination and communication among health professionals, described as “the optimal care standard.”^6^ Benefits associated with MDM presentation include improved diagnostic accuracy,^7^ improved staging evaluation,^7^^,^^8^ improved access to treatment,^9^, ^10^, ^11^ increased provision of curative intent treatment and surgery,^12^^,^^13^ and may contribute positively to emotional support and physical well-being.^14^ Adherence to MDM recommendations encourages management concordance with international guidelines,^8^^,^^11^^,^^15^ improved timeliness to treatment,^8^^,^^16^ reduced length of hospital stay,^17^ increased clinical trial participation and increased referrals to palliative care,^8^^,^^12^^,^^18^, ^19^, ^20^ ultimately improving patient survival.^18^^,^^21^^,^^22^

Clinical quality registries (CQRs) systematically monitor the quality, appropriateness, and effectiveness of health care within specific clinical domains by routine collection and analysis of real-world clinical data to provide institutional benchmarking for clinically relevant quality measures.^23^^,^^24^ These benchmarked reports allow health care providers to evaluate their performance against best evidence-based practice, identify any inequity, disparity, and unwarranted clinical variation, and facilitate data-driven self-assessment to promote continuous improvement in healthcare quality, safety, and appropriateness of care.^25^ Benchmarked reporting provides identification of evidence-practice gaps, enabling clinicians and governance opportunities to innovate and implement solutions that optimize best evidence-based practice.^26^

Many MDM outcomes have been evaluated at the institutional level; however, there is a critical need to extend this research to evaluate hypothesis-driven impacts of MDM presentation. We sought to evaluate the impacts of the Victorian Lung Cancer Registry (VLCR) function on presentation to a lung cancer MDM in a state-based lung cancer population.^27^^,^^28^ We aimed to describe the characteristics of patients presented and not presented to the lung cancer MDM; to explore inequity, disparity, and unwarranted clinical variation; to identify barriers and enablers to presentation; and to evaluate the impacts of MDM presentation on patient management and survival.^19^

## Methods

### Study Design and Setting

This study was an observational cohort study, using prospectively collected data on consecutively registered patients in the VLCR between July 2011 and December 2023, with opt-out consent of less than 3%. The VLCR is a CQR that collects observational data on patients with a principal diagnosis of lung cancer identified through 51 participating hospitals in Victoria, Australia, including metropolitan, regional, public, and private hospitals.^28^ Patients are identified using hospital-coded admission data (International Statistical Classification of Diseases and Related Health Problems, tenth revision, Australian modification C34.0–C34.9, Z85.1, Z85.2) and medical record review conducted by trained data collectors.

### Participants

Eligible participants included consecutive patients aged 18 years or older with newly diagnosed primary lung cancer who were diagnosed, received treatment, or were admitted to a VLCR-participating hospital. Lung cancers included in the cohort were NSCLC, SCLC, and other lung cancers (carcinoid tumors, adenoid cystic carcinomas, lymphomas, sarcomas, and hamartomas). Patients with secondary lung cancer deposits were excluded. Invitation letters to participate in the VLCR were sent to all eligible participants using an opt-out consent process. Patients who chose to opt out were excluded from the registry.

### Bias

Selection bias was minimized by including all consecutive lung cancer diagnoses within the study period. Information and measurement bias were addressed by using a standardized data dictionary and robust data validation processes. Unmeasured confounding remains a potential source of bias, as the VLCR captures a finite data set and does not include patient characteristics such as frailty or treatment-related factors, including treatment tolerance, toxicity, radiation dose, fractionation, treatment sequencing, and treatment completion.

### Variables

#### Demographic Variables

Basic patient demographics were collected, including age (categorized in 10-year cohorts), sex, and Indigenous Australian status. Region of patient residence and treating hospital locations were defined using the Modified Monash Model (MM) of suburb and locality classification, containing seven categories between MM1 metropolitan and MM7 very remote communities.^29^ We modified this categorization to include MM1 metropolitan, MM2 regional centers, MM3 large regional towns, MM4 medium regional towns, and combined MM5 to MM7 as rural-remote centers, on the basis of the framework by the Australian Statistical Geography Standard–Remoteness Areas. Socioeconomic status was ranked on the basis of population-based quintiles using the Index of Relative Socioeconomic Disadvantage, with a score of one indicating greatest disadvantage and five indicating least disadvantage.^30^

#### Clinical Variables

Smoking status was categorized as never-smoker, ex-smoker, and current smoker. Performance status (PS) was defined using the Eastern Cooperative Oncology Group (ECOG) scale from score 0 (fully active) to score 4 (completely disabled).^31^ Comorbidities included the following: diabetes (defined as patients receiving insulin or oral hypoglycemic treatment); renal insufficiency (serum creatinine greater than 200 μmol/L or requiring dialysis); myocardial infarction (history of myocardial infarction or coronary artery intervention); respiratory comorbidity (forced expiratory volume <60% predicted); and neoplastic comorbidity (history of cancer other than lung cancer).

#### Cancer Variables

Cancer type was defined histologically as NSCLC, SCLC, or other lung cancer. Cancer staging was categorized using the tumor, nodes, and metastases (TNM 7–8) system, into Stage I, II, III, and IV.^32^^,^^33^

#### Hospital and Location Characteristics

Hospital status was categorized into administrative type (public or private) and location (metropolitan or regional). Public and private hospital management and survival outcomes have been identified and described.^34^ The treating hospital for each patient was defined as the hospital used for the first surgical, radiotherapy, or systemic anticancer therapy (SACT). Where these data were not available, or the treating hospital data were not sufficient to establish location, we used the notifying hospital (<2%).

Driving time from residence to treating hospital has been defined, on the basis of postcode location, in categories less than 1 hour, 1 to 3 hours, or more than 3 hours.^35^ For a small number of postcodes, travel time data was not provided, and so we used travel time from an adjacent postcode. A minimum travel time of 15 minutes was set, even if the patient lived in the same postcode as the hospital.

#### Management and Treatment Variables

Timeliness intervals included time from referral to diagnosis (target <28 d), time to first treatment from diagnosis (target <14 d), and time to first treatment from referral date (target <42 d). Management variables included discussion at an MDM, treatment, and molecular testing for programmed death-ligand 1, EGFR, ALK, and ROS1 status in patients with NSCLC.

Treatments recorded included surgical resection, SACT, and radiotherapy. Treatment provided was categorized into guideline-concordant treatment (GCT), non-GCT, or no treatment received for both NSCLC (Supplementary Table 1) and SCLC (Supplementary Table 2). GCT was defined as the minimal treatment patients should receive on the basis of the National Comprehensive Cancer Network guidelines.^3^^,^^36^, ^37^, ^38^, ^39^, ^40^ Non-GCT was defined as treatment inconsistent with the guidelines or incomplete treatments. No treatment was defined as no recorded treatment.

### Statistical Methods

Patient, disease, and management characteristics of MDM-presented versus MDM nonpresented patients were compared using the Wilcoxon rank sum test and Fisher's Exact Test with simulated *p* value (on the basis of 2000 replicates). Descriptive data were presented using the mean ± SD for numerical data and percentages for count data. The likelihood of MDM discussion was determined using logistic regression reported as OR and 95% confidence intervals (CI). MDM presentation and overall survival effects were then assessed in a Cox model including propensity scores as an additional covariate. To assess the relationship between MDM presentations and patient survival between subgroups, an interaction between MDM presentation and subgroup was fitted. Subgroups evaluated reflected clinical stage, ECOG PS, hospital location, and hospital type. Univariable and multivariable analyses for overall survival were performed using Cox proportional hazards regression with results presented as hazard ratios (HR) and 95% CI. Variables with *p* less than 0.05 on univariable analyses or those deemed to be clinically relevant were entered into a hierarchical regression model to assess the independent association between MDM presentation and overall survival.

Trend analysis in proportion of patients presented to MDM was assessed using Mann-Kendall trend analysis for monotonic change, assessing change in median attainment in MDM presentation and interquartile range over time.

Overall survival was calculated from the date of diagnosis until death or census date on 10 January 2024. The Kaplan–Meier product-limit method was used to plot survival as a function of time, and comparisons between survival curves were made through the log-rank test. Sensitivity analyses were conducted using landmark analysis and propensity score matching methods to account for confounding factors. A landmark analysis was conducted to account for early deaths that may have prevented MDM discussion, comparing median survival between MDM-presented and nonpresented groups, excluding patients who died within 4 weeks of diagnosis. Propensity scores were generated using a multivariable logistic regression model with MDM presentation as the dependent variable and baseline characteristics that were unbalanced between groups or had clinical relevance as the independent variables (age, sex, socioeconomic status, hospital driving time, ECOG PS, comorbidities, clinical stage, hospital location (metropolitan versus regional), and hospital type (public versus private). All standardized mean difference values for matched covariates were less than the recommended guidelines of 0.1.^41^ All calculated *p* values were two-tailed, and a *p* less than 0.05 indicated statistical significance. Statistical analyses were performed using R version 4.4.3 (R Core Team, The R Foundation for Statistical Computing, Vienna, Austria).

### Ethics Approval

Ethics approval for this study was obtained from the Monash University Human Research Ethics Committee (47393). Ethics approval for the VLCR was obtained at Alfred Health under the National Mutual Acceptance scheme with governance clearance across all participating health services.

## Results

### Patient Characteristics

The cohort included 18,597 patients, of whom 12,525 (67%) had evidence of presentation to a lung cancer MDM and 6072 (33%) had no evidence of presentation to an MDM (Table 1). Age at diagnosis was slightly younger in MDM-presented patients, with a mean of 69 (SD 11) versus 71 (SD 11) years (*p* < 0.001). Patients in the 80 to 89 and 90 years and older age categories were less likely to be presented to the MDM (*p* < 0.001). Males represented 55% of patients overall and were slightly more likely to be MDM-presented (*p* = 0.011). Current smokers (36.0%) were more likely to be MDM-presented than ex-smokers (30.4%, *p* < 0.001).

**Table 1:** Baseline Patient Characteristics for NSCLC, SCLC, and Other Thoracic Cancers

Variable	Overall N = 18,597	No MDM N = 6072 (33%)	MDM N = 12,525 (67%)	*p* Value
**Age at diagnosis (y)**	70 ± 11	71 ± 11	69 ± 11	**<0.001**
**Sex**				**0.011**
Male	10,305 (55.4%)	3284 (54.1%)	7021 (56.0%)	
Female	8292 (44.6%)	2788 (45.9%)	5504 (44.0%)	
**Indigenous status**				0.3
Nonindigenous	18,162 (98.9%)	5957 (32.8%)	12,205 (67.2%)	
Indigenous	193 (1.1%)	56 (29.0%)	137 (71.0%)	
Missing data	242 (1.3%)	59 (1.0%)	183 (1.5%)	
**Smoking status**				**<0.001**
Never	2321 (12.5%)	772 (12.7%)	1549 (12.4%)	
Ex-smoker	9346 (50.2%)	3113 (51.3%)	6233 (49.8%)	
Current smoker	6360 (34.2%)	1845 (30.4%)	4515 (36.0%)	
Missing data	570 (3.1%)	342 (5.6%)	228 (1.8%)	
**Residential status**				0.2
Metropolitan	12,677 (68.1%)	4071 (67.0%)	8606 (68.7%)	
Regional centers	1533 (8.2%)	502 (8.3%)	1031 (8.2%)	
Large regional towns	688 (3.7%)	234 (3.8%)	454 (3.6%)	
Medium regional towns	756 (4.1%)	254 (4.2%)	502 (4.0%)	
Rural and remote areas	2930 (15.7%)	1007 (16.6%)	1923 (15.3%)	
**Driving distance**				**0.037**
<1 h	14,637 (78.7%)	4709 (77.6%)	9928 (79.2%)	
1 to 3 h	2804 (15.1%)	958 (15.8%)	1846 (14.7%)	
>3 h	1152 (6.2%)	402 (6.6%)	750 (5.9%)	
**IRSAD quintile**				**<0.001**
1 (most disadvantaged)	3486 (18.7%)	1069 (17.6%)	2417 (19.3%)	
2	2514 (13.5%)	780 (12.8%)	1734 (13.8%)	
3	3773 (20.3%)	1133 (18.6%)	2640 (21.1%)	
4	3413 (18.3%)	1089 (17.9%)	2324 (18.6%)	
5 (most advantaged)	5401 (29.2%)	1996 (32.9%)	3405 (27.2%)	
**ECOG**				**<0.001**
0	4290 (23.1%)	802 (13.2%)	3488 (27.8%)	
1	5556 (29.9%)	1248 (20.6%)	4308 (34.4%)	
2	1937 (10.4%)	573 (9.4%)	1364 (10.9%)	
3	861 (4.6%)	401 (6.6%)	460 (3.7%)	
4	121 (0.6%)	71 (1.2%)	50 (0.4%)	
Missing data	5832 (31.1%)	2977 (49.0%)	2855 (22.8%)	
**Lung cancer type**				**<0.001**
NSCLC	15,739 (85%)	4820 (79.4%)	10,919 (87.2%)	
SCLC	2095 (11%)	918 (15.1%)	1177 (9.4%)	
Other lung cancer type	763 (4.1%)	334 (5.5%)	429 (3.4%)	
**Clinical stage**				**<0.001**
I	2701 (14.5%)	472 (7.8%)	2229 (17.8%)	
II	1355 (7.3%)	204 (3.4%)	1151 (9.2%)	
III	2752 (14.8%)	434 (7.1%)	2318 (18.5%)	
IV	7845 (42.2%)	3153 (51.9%)	4692 (37.5%)	
Missing data	3944 (21.2%)	1809 (29.7%)	2135 (17.0%)	
**Diabetes**	2922 (15.7%)	903 (14.9%)	2019 (16.1%)	**0.028**
**Renal insufficiency**	401 (2.2%)	145 (2.4%)	256 (2.0%)	0.130
**Myocardial infarction**	2794 (15.0%)	912 (15.0%)	1882 (15.0%)	>0.9
**Respiratory comorbidity**	4432 (23.8%)	1133 (18.6%)	3299 (26.3%)	**<0.001**
**Neoplastic comorbidity**	3810 (20.5%)	1191 (19.6%)	2619 (21.0%)	**0.040**
**No comorbidities**	8346 (44.8%)	2949 (48.6%)	5397 (43.1%)	**<0.001**
**Hospital type**				**<0.001**
Public	15,522 (83.4%)	4358 (71.8%)	11,164 (89.1%)	
Private	3075 (16.6%)	1714 (28.2%)	1361 (10.9%)	
**Hospital regional status**				**<0.001**
Metropolitan	15,948 (85.7%)	5101 (84.0%)	10,847 (86.6%)	
Regional	2649 (14.3%)	971 (16.0%)	1678 (13.4%)	

ECOG, Eastern Cooperative Oncology Group; IRSAD, Index of Relative Socioeconomic Advantage and Disadvantage; MDM, Multidisciplinary Meeting.

The lack of clinical documentation of key patient characteristics, including ECOG status (no MDM 49.0% versus MDM 22.8%) and clinical stage (no MDM 29.7% versus MDM 17.0%) were more evident in non-MDM versus MDM-presented patients. Patients with good ECOG PS (ECOG <2) were more likely to have MDM-presented (ECOG 0-1: MDM 62.2% versus no MDM 33.8%, *p* < 0.001). Cases of NSCLC were more likely to be presented to MDM than SCLC (87.2% versus 9.4%, *p* < 0.001). There were 21.2% of patients who had no stage documented, and patients with stage IV were less likely to be MDM-presented (stage IV: no MDM 51.9% versus 37.5% MDM, *p* < 0.001). Patients from metropolitan (68.0%) and public hospitals (71.9%) were more likely to be MDM-presented than regional and private hospitals (*p* < 0.001).

### Assessing Modifiable Barriers and Enablers to MDM Presentation Attainment

Univariable and multivariable regression analysis were performed to identify factors associated with increased or reduced likelihood (OR) of MDM presentation (Supplementary Table 3). Factors associated with an increased likelihood of MDM presentation in a multivariable model included lower socioeconomic class (Index of Relative Socioeconomic Disadvantage 2, OR 1.03 [1.01–1.06], *p* = 0.004), respiratory comorbidity (OR 1.06 [1.04–1.07], *p* < 0.001) and history of neoplastic disease (OR 1.02 [1.01–1.04], *p* = 0.004). Factors associated with a lower likelihood of MDM presentation in a multivariable model included increasing age with 80 to 89 year-olds 9% less likely and 90 year-olds and older 18% less likely to be presented, being ex-smokers, poorer ECOG PS (ECOG 2 OR 0.94 [0.92–0.97], *p* < 0.001), SCLC (OR 0.91 [0.89–0.93], *p* < 0.001), and higher stage disease (stage IV OR 0.81 [0.80–0.83], *p* < 0.001). A driving time of 1 to 3 hours and longer than 3 hours from residence to hospital was associated 3% reduction (*p* = 0.006) and 8% reduction (*p* < 0.001) in the likelihood of MDM presentation. Patients diagnosed in private hospitals were 22% less likely to be presented in an MDM (OR 0.78 [0.77–0.80], *p* < 0.001), and patients treated in regional hospitals 12% less likely (OR 0.88 [0.87–0.90], *p* < 0.001).

### Impacts of MDM on Management

All three timeliness intervals were less likely to be achieved in patients with NSCLC after MDM presentation. MDM-presented patients with NSCLC who had diagnosis to treatment interval of less than 14 days was 27.8% compared with 35.1% for nonpresented patients (*p* < 0.001) (Table 2). Among patients with NSCLC, those discussed at an MDM were more likely to receive GCT (56.2% versus 44.5%, *p* < 0.001) and non-GCT (20.9% versus 15.9%, *p* < 0.001) and less likely to receive no treatment (10.0% versus 21.4%) compared with nonpresented patients. MDM presentation was associated with considerably higher levels of surgical resection, radiotherapy, and SACT (*p* < 0.001), and associated with considerably higher levels of testing for EGFR, ALK, ROS1, and programmed death-ligand 1 alterations (*p* < 0.001). There were 86% of patient diagnoses that were achieved in metropolitan hospitals with slightly higher MDM presentation than regional hospitals (68.0% versus 63.3%, *p* < 0.001).

**Table 2:** NSCLC Management Outcomes Dependent on MDM Presentation

Variable	Overall N = 15,739	No MDM N = 4820 (31%)	Yes MDM N = 10,919 (69%)	*p* Value
Referral to diagnosis within 28 days^a^	9506 (60.4%)	3201 (66.4%)	6305 (57.7%)	**<0.001**
Diagnosis to treatment within 14 days^a^	4725 (30.0%)	1691 (35.1%)	3034 (27.8%)	**<0.001**
Referral to treatment within 42 days^a^	5326 (33.8%)	1910 (39.6%)	3416 (31.3%)	**<0.001**
GCT	8282 (52.4%)	2145 (44.5%)	6137 (56.2%)	**<0.001**
Non GCT	3055 (19.4%)	768 (15.9%)	2287 (20.9%)	
No treatment	2125 (13.5%)	1033 (21.4%)	1092 (10.0%)	
No stage assessed	2277 (14.5%)	874 (18.1%)	1403 (12.8%)	
Surgical resection	3762 (23.9%)	846 (17.6%)	2916 (26.7%)	**<0.001**
SACT	7448 (47.3%)	2195 (45.5%)	5253 (48.1%)	**0.003**
Radiotherapy	7094 (45.1%)	1966 (41.4%)	5128 (47.0%)	**<0.001**
EGFR tested	6046 (38.4%)	1617 (33.5%)	4429 (40.6%)	**<0.001**
ALK tested	5149 (32.7%)	1484 (30.8%)	3665 (33.6%)	**<0.001**
ROS1 tested	1810 (11.5%)	485 (10.1%)	1325 (12.1%)	**<0.001**
PDL1 tested	8084 (51.4%)	2000 (41.5%)	6084 (55.7%)	**<0.001**

GCT, guideline-concordant treatment; PD-L1, programmed death ligand 1; SACT, systemic anticancer treatment.

a Variables include missing values.

### Impacts of MDM Quality Attainment and Clinical Covariates on Mortality Hazard

MDM presentation was associated with a 25% reduction in mortality hazard (HR 0.75 [0.72–0.78], *p* < 0.001) (Supplementary Table 4). Females had a 18% lower mortality hazard compared with males (HR 0.82 [0.80–0.86], *p* < 0.001). Factors associated with increased mortality included increasing age (70–79 y HR 1.28 [1.2–1.4], *p* < 0.001), current smokers (HR 1.68 [1.6–1.8], *p* < 0.001), ex–smokers (HR 1.48 [1.4–1.6], *p* < 0.001), poorer ECOG PS (ECOG 2 HR 2.0 [1.9–2.1], *p* < 0.001), increasing clinical stage (stage II HR 1.78 [1.6–2.0], *p* < 0.001), SCLC (HR 1.44 [1.4–1.5], *p* < 0.001), renal insufficiency (HR 1.20 [1.1–1.3], *p* = 0.002) and neoplastic comorbidities (HR 1.06 [1–1.1], *p* = 0.023), and regional and remote residential location (medium regional towns HR 1.26 [1.1–1.4], *p* < 0.001). Private hospital management was associated with a 30% reduction in mortality hazard (HR 0.70 [0.66–0.75], *p* < 0.001).

### Temporal Trends in MDM Presentation and Unwarranted Clinical Variation

**Figure 1:**
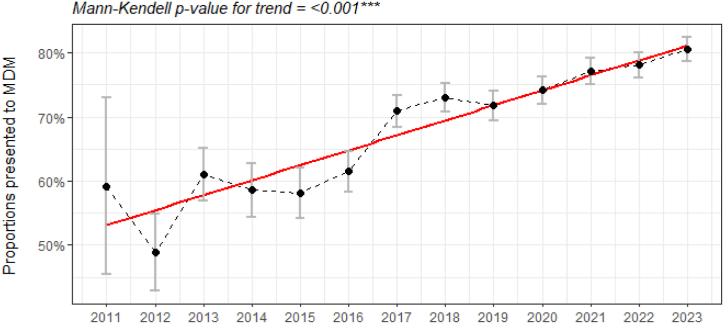
Proportion of patients with presentation at a lung cancer MDM documented (n = 18,597, Mann-Kendall *p* < 0.001). MDM, multidisciplinary meeting.

There has been an increase in MDM quality indicator (QI) attainment over time at the total cohort level (Fig. 1). The proportion of patients presented to the MDM in 2011 was 59.1%, 71% in 2017, and 80.6% in 2023. Mann-Kendall trend analysis revealed a considerable increase in MDM-presented proportions (*p* < 0.001).

**Figure 2:**
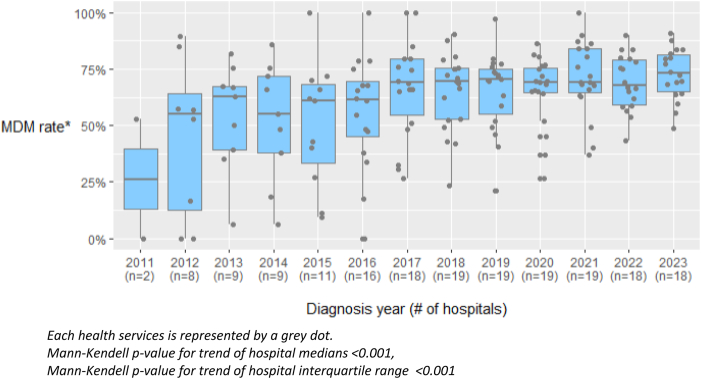
Change in unwarranted clinical variation across health services for MDM presentation. MDM, multidisciplinary meeting.

Over time, there has been a reduction in unwarranted clinical variation across health services (Fig. 2). There was a considerable increase in the hospital median proportions of patients presented to the MDM (Mann-Kendell trend analysis, *p* < 0.001), and a decrease in the hospital interquartile ranges over time (Mann-Kendell trend analysis, *p* < 0.001).

### Impacts on Survival Benefit

Overall survival for the total NSCLC cohort (n = 15,737) revealed a median survival of 2.2 years (2.1–2.3 y) for MDM-presented patients compared with 0.94 years for nonpresented patients (CI 0.88–1.0, *p* <0.001), providing a 34% reduction in mortality hazard (HR 0.66 [0.63–0.69], *p* < 0.001). For the SCLC cohort (n = 2094), MDM-presented patients had a median survival of 0.94 years compared with 0.57 years for nonpresented patients (HR 0.66 [0.60–0.73], *p* < 0.001).

Landmark survival analysis of 14,931 patients with NSCLC (excluding 808 patients who died within 28 days of diagnosis), revealed a median survival of 2.3 years (2.3–2.5 y, *p* < 0.001), compared with nonpresented patients 1.2 years (1.1–1.3 y), providing a 29% reduction in mortality hazard (HR 0.71 [0.68–0.74], *p* < 0.001). Landmark survival analysis of 1881 patients with SCLC (excluding 214 patients who died within 28 days of diagnosis) revealed a median survival of 0.99 years (0.94–1.1 y, *p* < 0.001), compared with 0.69 years for nonpresented patients (0.65–0.77 y), providing a 28% reduction in mortality hazard (HR 0.72 [0.65–0.80], *p* < 0.001).

### Propensity-Matched Survival Analysis

**Figure 3:**
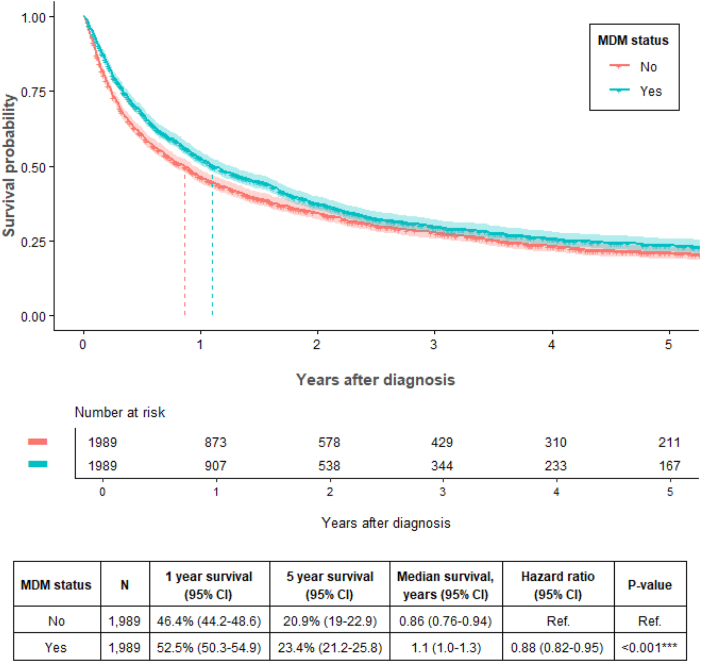
Propensity-matched survival outcomes for all-stage NSCLC MDM-presented (n = 1989) and nonpresented patients with lung cancer (n = 1989). Propensity matching included the following variables: age, sex, socioeconomic status, hospital driving time, ECOG PS, comorbidities, clinical stage, hospital location (metropolitan versus regional), and hospital type (public versus private). MDM, multidisciplinary meeting; ECOG PS, Eastern Cooperative Oncology Group performance status; CI, confidence interval.

Propensity-matched survival analysis for all-stage NSCLC (n = 3978) identified a median survival for MDM-presented patients of 1.1 years (1.0–1.3 y) versus 0.86 years (0.76–0.94 y) with a reduction in mortality hazard of 12% (HR 0.88 [0.82–0.95], *p* < 0.001) (Fig. 3). Propensity-matched survival for stage IV NSCLC (n = 3028) identified a median survival for MDM-presented patients of 0.77 years (0.65–0.85 y) compared with 0.54 years (0.47–0.60 y) for non–MDM-presented patients, with an 18% reduction in mortality hazard (HR 0.82 [0.76–0.89], *p* < 0.001) (Supplementary Fig. 1). Propensity-matched survival analysis for all-stage SCLC (n = 500) identified a median survival for MDM-presented patients of 0.67 years (0.60–0.74 y) versus 0.61 years (0.53–0.75 y) with a not considerable reduction in mortality hazard of 7% (HR 0.93 [0.77–1.13], *p* = 0.5) (Supplementary Fig. 2).

## Discussion

We observed a marked increase in the proportion of patients presented to an MDM over the reporting period of the VLCR, rising from 59.1% in 2011 to 80.6% in 2023, associated with a reduction in unwarranted clinical variation in MDM presentation between participating hospitals. We observed a 34% reduction in overall mortality hazard in association with MDM presentation, and 12% reduction in propensity-matched analysis for patients with NSCLC presented to a lung cancer MDM. Clinical documentation of ECOG and clinical stage, which enable informed decision making in lung cancer management, was doubled in MDM versus non–MDM-presented patients. Patients with stage IV lung cancer were least likely to be presented to the MDM (59.8%), and yet stage IV NSCLC experienced an 18% reduction in propensity-matched mortality hazard after MDM presentation. Cox regression analysis identified multiple modifiable factors associated with nonpresentation, including increased age, poorer ECOG status, clinical stage, smoking status, hospital location, and hospital administration status.

A common MDM format involves clinicians meeting weekly or fortnightly to decide on optimum investigation and management plans in suspected or confirmed lung cancer cases. This format poses resource, personnel, and logistical challenges for institutions, and clinician availability is likely to influence MDM quality and effectiveness.^42^ Practical barriers, such as administrative support, facilities, resources, data capture, storage, and communication, may impact accessibility and quality of MDMs in some hospitals and limit the accuracy, completeness, and utilization of collected data.^43^ More evidence is needed to explore patient, disease, and hospital characteristics that drive MDM presentation, identifying potentially modifiable factors and clinically relevant outcomes.^35^ Clarifying these associations may enable clinicians and hospitals to inform and evaluate MDM referral and utilization patterns and to identify targets for improvement in MDM function.

### Modifiable Factors in MDM Presentation

Therapeutic nihilism is a potential barrier to MDM presentation. Referring clinicians may limit MDM referrals in older patients because of concerns related to age, frailty, or treatment tolerability, which may be influenced by nihilistic attitudes.^43^ With a mean age of 70 years at diagnosis, some 35% of patients are aged 70 to 79, and 19% aged above 80 years. We observed that 31.0% of patients aged 70 to 79 years and 40.3% of patients aged 80 to 89 years were not referred for MDM discussion. Comprehensive geriatric assessment in oncology has been recommended for patients older than 70 years (comprising 50% of patients with lung cancer), revealing that nearly half of the recommended treatment plans may need modifications to optimize treatment delivery.^37^^,^^44^

Patients with stage IV disease had a substantial reduction in the rates of MDM presentation, highlighting the need for closer assessment of stage-specific impacts of MDM presentation. Finally, differential work practices between public and private hospitals pose additional challenges to the coordination of MDM, which suggests the need for increased collaboration between policymakers, hospital governance, and clinicians.

### Registry Causation or Association in Indicator Improvement?

Assessing the impact of CQR on clinical QI attainment is challenging. Although any observed change in clinical QI attainment during the registry era is a reflection of association, evidence of causation for the role of CQR in quality improvement in disease management is difficult to establish. A critical barrier to this assessment is the lack of clinical data, such as clinical stage and PS, from administrative health care data sets before the establishment of registries, making before-and-after evaluations rarely possible.

### Further Opportunities for CQR Data

CQRs have clear and established roles in the delivery of risk-adjusted, benchmarked indicator reports to stakeholder institutions and the broader lung cancer community. These reports enable context-specific evaluation of clinical performance that targets quality improvement,^45^, ^46^, ^47^, ^48^ supports longitudinal reporting of QI and service redesign initiatives,^45^^,^^49^ describes unwarranted clinical variation in clinical process and outcomes,^50^ and describes variation in access and equity in health care^20^^,^^43^ in a sustainable and cost-effective manner.^51^

Ouwens et al.^52^ reported on the use of the Dutch Lung Cancer Registry, reporting multidisciplinary consultation in 57% of patients evaluated (range 26%–91%). Recent meta-analysis in NSCLC has found an overall 37% reduction in mortality hazard for those presented to lung cancer MDMs, demanding further evaluation of stage-specific survival outcomes.^19^

### Learning Health Systems

Learning health systems combine different types of information, enabling improvement collaboratives to find strategies to integrate and apply this knowledge to enable better decision making, creating better health care outcomes, and addressing evidence-practice gaps.^53^, ^54^, ^55^, ^56^ The learning health systems incorporate three important information sources. First is the knowledge that tells us “what we should be doing” for our patients in terms of best evidence-based practice^57^ and clinical practice guidelines.^25^ Second, the knowledge that tells us “what we are actually doing,”—that is, clinical performance quality as measured by CQRs. And third is the evaluation and learning from the combinations of these two knowledge sources that enable us to “plan, innovate, and implement for better outcomes.”

### Strengths and Limitations

This article has a major strength in the use of a single, population-based data infrastructure collecting data to describe disease outcomes for all consecutively diagnosed patients living with lung cancer in Victoria, Australia. There is likely to be some selection bias among patients because of the nonparticipation of some private health networks and probable under representation of those diagnosed in regional and remote Victoria. The VLCR has a parsimonious data collection strategy because of cost limitations restricted to a limited number of QIs.^27^ The registry, therefore, describes first assessment and treatment but does not describe second and subsequent lines of treatment and lung MDM assessment for these time points.

A further limitation is unmeasured confounding with considerable missing data in areas such as ECOG and stage, and additional measures reflecting patient frailty and comorbidity. Sensitivity analyses partially address this issue and identify reduced mortality hazard in selected cohorts. In addition, there were considerable survival differences between patients treated in private and public hospitals, potentially reflecting unmeasured differences in treatment access, socioeconomic disadvantage, adverse exposures, personal habits, physical activity, health maintenance, health-seeking, and treatment behaviors.^34^^,^^58^

### Generalizability

This large and contemporary study of 18,597 Victorian people with lung cancer provides findings that are likely to be applicable to Australian states and territories, given similar populations, health service infrastructure, and health care implementation. The international acceptance of the definition of multidisciplinary meeting structure and process provides some optimism for confirmation of impacts globally, which may support international generalizability.

In conclusion, the VLCR identified and measured MDM presentation as a key QI reflecting the quality of delivered best evidence-based practice care. During the operation of the VLCR, there is evidence of an increased overall rate of MDM discussion regarding diagnosis and management plans for patients with lung cancer, a reduction in unwarranted variation across health services, improvements in patterns of delivered care, and an association with a reduction in mortality hazard for those discussed in lung cancer MDMs.

## CRediT Authorship Contribution Statement

**Rob G Stirling:** Conceptualization, Methodology, Validation, Formal analysis, Investigation, Data curation, Writing - original draft, Writing - review & editing, Visualization, Supervision, Project administration.

**Sanuki Tissera:** Conceptualization, Methodology, Validation, Formal analysis, Investigation, Data curation, Writing - original draft, Writing - review & editing, Visualization, Supervision, Project administration.

**Jessie Zeng:** Writing - original draft, Writing - review & editing, Visualization.

**Mike Lloyd:** Conceptualization, Methodology, Validation, Formal analysis, Investigation, Data curation, Writing - original draft, Writing - review & editing, Visualization, Supervision, Project administration.

**Krupa Krishnaprasad:** Conceptualization, Methodology, Validation, Formal analysis, Investigation, Data curation, Writing - review & editing, Supervision, Project administration.

**Lisa Briggs:** Validation, Investigation, Writing - review & editing.

**Jacqueline Lesage:** Validation, Investigation, Writing - review & editing.

**Tom Wood:** Validation, Investigation, Writing - review & editing.

**Craig Underhill:** Validation, Investigation, Writing - review & editing.

**Sagun Parakh:** Validation, Investigation, Writing - review & editing.

**Louis Irving:**

**Wasek Faisal:** Validation, Investigation, Writing - review & editing.

**Robert Blum:** Writing - review & editing

**Gary Richardson:** Writing - review & editing

**Phillip Parente:** Validation, Investigation, Writing - review & editing.

**Michelle Caldecott:** Validation, Investigation, Writing - review & editing, Writing - review & editing.

**Inger Olesen:** Validation, Investigation, Writing - review & editing.

**Javier Torres:** Validation, Investigation, Writing - review & editing.

**Evangeline Samuel:** Validation, Investigation, Writing - review & editing.

**Christopher Lyne:** Validation, Investigation, Writing - review & editing.

**Katharine See:** Validation, Investigation, Writing - review & editing.

**David Langton:** Validation, Investigation, Writing - review & editing.

**Tom John:** Validation, Investigation, Writing - review & editing.

**Gavin M Wright:** Validation, Investigation, Writing - review & editing.

**Matthew Conron:** Validation, Investigation.

**James Bartlett:** Validation, Investigation, Writing - review & editing.

**Nicola Atkin:** Validation, Investigation, Writing - review & editing.

**Maggie Moore:** Conceptualization, Methodology, Validation, Investigation, Writing - review & editing.

**Golsa Adabi:** Conceptualization, Methodology, Validation, Investigation, Writing - review & editing.

**Luke Clegg:** Validation, Investigation, Writing - review & editing.

**Nikolajs Zeps:** Conceptualization, Methodology, Validation, Investigation, Writing - review & editing, Project administration.

**Susan Harden:** Validation, Investigation, Writing - review & editing.

**Zoe McQuilten:** Conceptualization, Methodology, Validation, Investigation, Writing - review & editing.

**John Zalcberg:** Conceptualization, Methodology, Validation, Formal analysis, Investigation, Visualization, Supervision, Project administration.

**LL:** Validation, Investigation, Writing - original draft, Writing - review & editing.

## Disclosure

The authors declare no conflicts of interest.
