# Supplementary material for: Impacts of Multidisciplinary Lung Cancer Meeting Presentation in a Clinical Quality Registry
Source: JTO Clin Res Rep. 2026 Mar 6;7(5):100984. doi: 10.1016/j.jtocrr.2026.100984 (PMC13089129; doi:10.1016/j.jtocrr.2026.100984)
Supplement: Supplementary Table 1 [file mmc1.docx]

**Supplementary Table 1.** Definition of NSCLC guideline-concordant treatment (40).

| Disease | Clinical stage | Definitions of guideline-concordant (GCT) and non-guideline concordant treatment (non-GCT) |
| --- | --- | --- |
| NSCLC | Stage I | **GCT:**  Surgery and/or SABR^1^ ± additional treatments |
|  |  | **Non-GCT:**  Treatments not involving surgery or SABR |
|  | Stage II | **GCT:**  Surgery + SACT^2^ ± additional treatments |
|  |  | **Non-GCT:**  Treatments not involving a combination of surgery + SACT^2^ |
|  | Stage III | **GCT:**  Radiotherapy + SACT^2^ ± additional treatments; or  Surgery + SACT^2^ ± additional treatments |
|  |  | **Non-GCT:**  Any treatments not involving SACT^2^ in combination with surgery or radiotherapy |
|  | Stage IV | **GCT:**  SACT^2^ ± additional treatments |
|  |  | **Non-GCT:**  Treatments not involving SACT^2^ |
| *^1^ Stereotactic ablative body radiotherapy, ^2^ Systemic anti-cancer therapy* | | |
