# Supplementary material for: Impacts of Multidisciplinary Lung Cancer Meeting Presentation in a Clinical Quality Registry
Source: JTO Clin Res Rep. 2026 Mar 6;7(5):100984. doi: 10.1016/j.jtocrr.2026.100984 (PMC13089129; doi:10.1016/j.jtocrr.2026.100984)
Supplement: Supplementary Table 2 [file mmc2.docx]

**Supplementary Table 2**. Definition of SCLC guideline concordant treatment (38).

| Disease | Clinical stage | Definitions of guideline-concordant (GCT) and non-guideline concordant treatment (non-GCT) |
| --- | --- | --- |
| SCLC | Limited Stage-Stage I-III SCLC | **GCT:**  Surgery and SACT^1^  Surgery, SACT and radiotherapy  SACT and radiotherapy |
|  |  | **Non-GCT:**  Surgery and radiotherapy  Surgery only  SACT only  Radiotherapy only |
|  | Extensive stage-Stage IV SCLC | **GCT:**  SACT only  Surgery and SACT  Surgery, SACT and radiotherapy  SACT and radiotherapy |
|  |  | **Non-GCT**:  Surgery and radiotherapy  Surgery only  Radiotherapy only |
| *^1^SACT = Systemic anti-cancer therapy which included chemotherapy, immunotherapy and/or targeted therapy* | | |
