# Supplementary material for: Impacts of Multidisciplinary Lung Cancer Meeting Presentation in a Clinical Quality Registry
Source: JTO Clin Res Rep. 2026 Mar 6;7(5):100984. doi: 10.1016/j.jtocrr.2026.100984 (PMC13089129; doi:10.1016/j.jtocrr.2026.100984)
Supplement: Supplementary Table 3 [file mmc3.docx]

**Supplementary Table 3.** Univariable and multivariable regression analysis of factors associated with MDM presentation (NSCLC and SCLC).

| **Variable** | **Category** | **Univariable** | | | **Multivariable** | | |
| --- | --- | --- | --- | --- | --- | --- | --- |
|  |  | **N** | **OR (95% CI)** | **p-value** | **N** | **OR (95% CI)** | **p-value** |
| Age | < 50 | 776 | Ref |  | 755 | Ref |  |
|  | 50-59 | 2,319 | 0.82 (0.68-0.99) | 0.036 | 2,272 | 0.96 (0.93-1.00) | 0.025 |
|  | 60-69 | 5,378 | 0.78 (0.66-0.93) | 0.005 | 5,254 | 0.96 (0.93-0.99) | 0.009 |
|  | 70-79 | 6,362 | 0.79 (0.67-0.94) | 0.007 | 6,176 | 0.96 (0.93-1.00) | 0.031 |
|  | 80-89 | 2,772 | 0.55 (0.46-0.66) | <0.001 | 2,646 | 0.91 (0.88-0.94) | <0.001 |
|  | 90 and over | 227 | 0.22 (0.16-0.30) | <0.001 | 204 | 0.82 (0.76-0.87) | <0.001 |
| Sex | Male | 9,907 | Ref |  |  |  |  |
|  | Female | 7,927 | 0.92 (0.86-0.98) | 0.007 |  |  |  |
| Indigenous status | Not Indigenous | 17,416 | Ref |  |  |  |  |
|  | Indigenous | 186 | 1.17 (0.85-1.62) | 0.3 |  |  |  |
| Smoking status | Never | 2,185 | Ref |  | 2,183 | Ref |  |
|  | Ex | 8,975 | 1.01 (0.91-1.12) | 0.8 | 8,971 | 0.98 (0.96-1.00) | 0.018 |
|  | Current | 6,156 | 1.23 (1.11-1.37) | <0.001 | 6,153 |  |  |
| Residential status | Metropolitan | 12,184 | Ref |  |  |  |  |
|  | Regional centers | 1,436 | 0.98 (0.87-1.10) | 0.8 |  |  |  |
|  | Large regional towns | 660 | 0.89 (0.75-1.05) | 0.2 |  |  |  |
|  | Medium regional towns | 728 | 0.94 (0.80-1.10) | 0.4 |  |  |  |
|  | Small - very remote areas | 2,814 | 0.91 (0.83-0.99) | 0.025 |  |  |  |
| Driving distance | <1 hour | 14,036 | Ref |  | 13,627 | Ref |  |
|  | 1 to 3 hours | 2,705 | 0.90 (0.83-0.98) | 0.019 | 2,616 | 0.97 (0.95-0.99) | 0.006 |
|  | >3 hours | 1,089 | 0.86 (0.75-0.97) | 0.018 | 1,064 | 0.92 (0.90-0.95) | <0.001 |
| IRSAD quintile | 1 | 3,361 | Ref |  | 3,297 | Ref |  |
|  | 2 | 2,405 | 0.98 (0.87-1.09) | 0.7 | 2,321 | 1.03 (1.01-1.06) | 0.004 |
|  | 3 | 3,611 | 1.04 (0.94-1.15) | 0.5 | 3,506 |  |  |
|  | 4 | 3,288 | 0.93 (0.84-1.03) | 0.2 | 3,206 |  |  |
|  | 5 | 5,160 | 0.74 (0.68-0.82) | <0.001 | 4,977 |  |  |
| ECOG | 0 | 4,161 | Ref |  | 4,128 | Ref |  |
|  | 1 | 5,399 | 0.79 (0.72-0.88) | <0.001 | 5,302 |  |  |
|  | 2 | 1,871 | 0.57 (0.50-0.64) | <0.001 | 1,832 | 0.94 (0.92-0.97) | <0.001 |
|  | 3 | 788 | 0.29 (0.25-0.34) | <0.001 | 764 | 0.84 (0.81-0.86) | <0.001 |
|  | 4 | 110 | 0.17 (0.12-0.25) | <0.001 | 108 | 0.75 (0.70-0.82) | <0.001 |
|  | Missing | 5,505 | 0.23 (0.20-0.25) | <0.001 | 5,173 | 0.79 (0.77-0.80) | <0.001 |
| Lung cancer type | NSCLC | 15,739 | Ref |  | 15,267 | Ref |  |
|  | SCLC | 2,095 | 0.57 (0.52-0.62) | <0.001 | 2,040 | 0.91 (0.89-0.93) | <0.001 |
| Clinical stage | I | 2,582 | Ref |  | 2,548 | Ref |  |
|  | II | 1,294 | 1.20 (1.00-1.45) | 0.047 | 1,281 |  |  |
|  | III | 2,670 | 1.15 (0.99-1.33) | 0.060 | 2,627 |  |  |
|  | IV | 7,570 | 0.32 (0.29-0.36) | <0.001 | 7,339 | 0.81 (0.80-0.83) | <0.001 |
|  | Missing | 3,718 | 0.25 (0.22-0.28) | <0.001 | 3,512 | 0.82 (0.81-0.84) | <0.001 |
| Diabetes | No | 15,020 | Ref |  | 14,564 | Ref |  |
|  | Yes | 2,814 | 1.11 (1.02-1.21) | 0.017 | 2,743 | 1.02 (1.00-1.03) | 0.071 |
| Renal | No | 17,451 | Ref |  |  |  |  |
|  | Yes | 383 | 0.88 (0.71-1.09) | 0.2 |  |  |  |
| Myocardial | No | 15,171 | Ref |  | 14,708 | Ref |  |
|  | Yes | 2,663 | 1.05 (0.96-1.15) | 0.3 | 2,599 | 1.01 (1.00-1.03) | 0.2 |
| Respiratory | No | 13,626 | Ref |  | 13,163 | Ref |  |
|  | Yes | 4,208 | 1.65 (1.53-1.79) | <0.001 | 4,144 | 1.06 (1.04-1.07) | <0.001 |
| Neoplastic | No | 14,185 | Ref |  | 13,769 | Ref |  |
|  | Yes | 3,649 | 1.08 (1.00-1.16) | 0.067 | 3,538 | 1.02 (1.01-1.04) | 0.004 |
| No comorbidities | No | 9,814 | Ref |  |  |  |  |
|  | Yes | 8,020 | 0.78 (0.73-0.83) | <0.001 |  |  |  |
| Hospital type | Public | 14,914 | Ref |  | 14,526 | Ref |  |
|  | Private | 2,920 | 0.30 (0.27-0.32) | <0.001 | 2,781 | 0.78 (0.77-0.80) | <0.001 |
| Hospital regional status | Metropolitan | 15,317 | Ref |  | 14,884 | Ref |  |
|  | Regional | 2,517 | 0.84 (0.77-0.92) | <0.001 | 2,423 | 0.88 (0.87-0.90) | <0.001 |
| *MDM: Multi-Disciplinary Meeting, IRSAD: Index of Relative Socio-economic Advantage and Disadvantage, ECOG: Eastern Co-operative Oncology Group, NSCLC: Non-Small Cell Lung Cancer, SCLC: Small Cell Lung Cancer* | | | | | | | |
