# Supplementary material for: Impacts of Multidisciplinary Lung Cancer Meeting Presentation in a Clinical Quality Registry
Source: JTO Clin Res Rep. 2026 Mar 6;7(5):100984. doi: 10.1016/j.jtocrr.2026.100984 (PMC13089129; doi:10.1016/j.jtocrr.2026.100984)
Supplement: Supplementary Table — 4 [file mmc4.docx]

**Supplementary Table 4.** Univariable and multivariable analysis of factors impacting mortality hazard (NSCLC and SCLC).

| **Variable** | **Category** | **Univariable** | | | **Multivariable** | | |
| --- | --- | --- | --- | --- | --- | --- | --- |
|  |  | **N** | **HR (95% CI)** | **p-value** | **N** | **HR (95% CI)** | **p-value** |
| MDM | No | 6,071 | Ref |  | 5,429 | Ref |  |
|  | Yes | 12,523 | 0.62 (0.6-0.65) | <0.001 | 11,874 | 0.75 (0.72-0.78) | <0.001 |
| Sex | Male | 10,303 |  |  | 9,670 |  |  |
|  | Female | 8,291 | 0.75 (0.72-0.78) | <0.001 | 7,633 | 0.82 (0.80-0.86) | <0.001 |
| Age category | < 50 | 824 | Ref |  | 755 | Ref |  |
|  | 50-59 | 2,400 | 1.12 (1-1.2) | 0.041 | 2,272 |  |  |
|  | 60-69 | 5,540 | 1.2 (1.1-1.3) | <0.001 | 5,253 | 1.12 (1-1.2) | 0.025 |
|  | 70-79 | 6,595 | 1.31 (1.2-1.4) | <0.001 | 6,175 | 1.28 (1.2-1.4) | <0.001 |
|  | 80-89 | 2,963 | 1.92 (1.7-2.1) | <0.001 | 2,644 | 1.75 (1.6-2) | <0.001 |
|  | 90 and over | 272 | 3.48 (3-4.1) | <0.001 | 204 | 2.61 (2.2-3.1) | <0.001 |
| Indigenous status | Not Indigenous | 18,159 | Ref |  |  | Ref |  |
|  | Indigenous | 193 | 1.03 (0.86-1.2) | 0.7 |  |  |  |
|  | Missing | 242 | 0.90 (0.76-1.1) | 0.2 |  |  |  |
| Smoking status | Never smoked | 2,321 | Ref |  | 2,182 | Ref |  |
|  | Ex-smoker | 9,345 | 1.58 (1.5-1.7) | <0.001 | 8,967 | 1.48 (1.4-1.6) | <0.001 |
|  | Current smoker | 6,360 | 1.77 (1.7-1.9) | <0.001 | 6,154 | 1.68 (1.6-1.8) | <0.001 |
| Residential status | Metropolitan | 12,675 | Ref |  | 11,826 | Ref |  |
|  | Regional centres | 1,532 | 0.93 (0.88-1) | 0.063 | 1,389 |  |  |
|  | Large regional towns | 688 | 0.87 (0.79-0.96) | 0.006 | 642 |  |  |
|  | Medium regional towns | 756 | 1.08 (0.99-1.2) | 0.080 | 715 | 1.26 (1.1-1.4) | <0.001 |
|  | Small - very remote areas | 2,930 | 0.98 (0.94-1) | 0.5 | 2,731 | 1.09 (1-1.2) | 0.018 |
| Driving distance | <1 hour | 14,634 | Ref |  | 13,626 | Ref |  |
|  | 1 to 3 hours | 2,804 | 0.98 (0.94-1) | 0.6 | 2,612 |  |  |
|  | >3 hours | 1,152 | 0.52 (0.48-0.58) | <0.001 | 1,065 | 0.64 (0.58-0.71) | <0.001 |
| IRSAD quintile | 1 | 3,486 | Ref |  |  |  |  |
|  | 2 | 2,514 | 0.97 (0.91-1) | 0.4 |  |  |  |
|  | 3 | 3,772 | 0.97 (0.92-1) | 0.5 |  |  |  |
|  | 4 | 3,412 | 0.97 (0.92-1) | 0.4 |  |  |  |
|  | 5 | 5,400 | 0.98 (0.94-1) | 0.6 |  |  |  |
| Lung cancer type | NSCLC | 15,737 | Ref |  | 15,262 |  |  |
|  | SCLC | 2,094 | 1.79 (1.7-1.9) | <0.001 | 2,041 | 1.44 (1.4-1.5) | <0.001 |
| Clinical stage | I | 2,701 | Ref |  | 2,546 | Ref |  |
|  | II | 1,355 | 1.9 (1.7-2.1) | <0.001 | 1,281 | 1.78 (1.6-2.0) | <0.001 |
|  | III | 2,752 | 3.11 (2.9-3.4) | <0.001 | 2,626 | 2.82 (2.6-3.1) | <0.001 |
|  | IV | 7,844 | 6.4 (5.9-6.9) | <0.001 | 7,338 | 5.67 (5.2-6.1) | <0.001 |
|  | Missing | 3,942 | 3.07 (2.8-3.3) | <0.001 | 3,512 | 2.51 (2.3-2.7) | <0.001 |
| ECOG | 0 | 4,290 | Ref |  | 4,127 | Ref |  |
|  | 1 | 5,556 | 1.71 (1.6-1.8) | <0.001 | 5,299 | 1.4 (1.3-1.5) | <0.001 |
|  | 2 | 1,936 | 2.76 (2.6-2.9) | <0.001 | 1,831 | 2.0 (1.9-2.1) | <0.001 |
|  | 3 | 861 | 4.79 (4.4-5.2) | <0.001 | 764 | 3.07 (2.8-3.4) | <0.001 |
|  | 4 | 121 | 10.3 (8.6-12) | <0.001 | 108 | 7.31 (6.0-8.9) | <0.001 |
|  | Missing | 5,830 | 1.63 (1.5-1.7) | <0.001 | 5,174 | 1.5 (1.4-1.6) | <0.001 |
| Diabetes | No | 15,672 | Ref |  | 14,560 |  |  |
|  | Yes | 2,922 | 1.18 (1.1-1.2) | <0.001 | 2,743 |  |  |
| Renal | No | 18,193 | Ref |  | 16,933 | Ref |  |
|  | Yes | 401 | 1.61 (1.4-1.8) | <0.001 | 370 | 1.2 (1.1-1.3) | 0.002 |
| Myocardial | No | 15,801 | Ref |  | 14,705 |  |  |
|  | Yes | 2,793 | 1.21 (1.2-1.3) | <0.001 | 2,598 |  |  |
| Respiratory | No | 14,163 | Ref |  | 13,160 |  |  |
|  | Yes | 4,431 | 1.11 (1.1-1.2) | <0.001 | 4,143 |  |  |
| Neoplastic | No | 14,785 | Ref |  | 13,767 | Ref |  |
|  | Yes | 3,809 | 0.96 (0.92-1) | 0.077 | 3,536 | 1.06 (1.0-1.1) | 0.023 |
| No comorbidities | No | 10,249 | Ref |  |  |  |  |
|  | Yes | 8,345 | 0.88 (0.85-0.92) | <0.001 |  |  |  |
| Hospital type | Public | 15,519 | Ref |  | 14,521 | Ref |  |
|  | Private | 3,075 | 0.69 (0.66-0.73) | <0.001 | 2,782 | 0.70 (0.66-0.75) | <0.001 |
| Hospital regional status | Metropolitan | 15,946 | Ref |  |  |  |  |
|  | Regional | 2648 | 1.29 (1.2-1.4) | <0.001 |  |  |  |
| *MDM: Multi-Disciplinary Meeting, IRSAD: Index of Relative Socio-economic Advantage and Disadvantage, ECOG: Eastern Co-operative Oncology Group, NSCLC: Non-Small Cell Lung Cancer, SCLC: Small Cell Lung Cancer* | | | | | | | |
