# Supplementary material for: Impacts of Multidisciplinary Lung Cancer Meeting Presentation in a Clinical Quality Registry
Source: JTO Clin Res Rep. 2026 Mar 6;7(5):100984. doi: 10.1016/j.jtocrr.2026.100984 (PMC13089129; doi:10.1016/j.jtocrr.2026.100984)

**Supplementary Figure 2.** Propensity matched survival outcomes for all stage SCLC Multi-Disciplinary Meeting-presented (n=250) and non-presented (n=250) patients (n=500). Propensity matching included the following variables (age, gender, socio-economic status, hospital driving time, ECOG PS, comorbidities, clinical stage, hospital location (metropolitan vs regional) and hospital type (public vs private)).


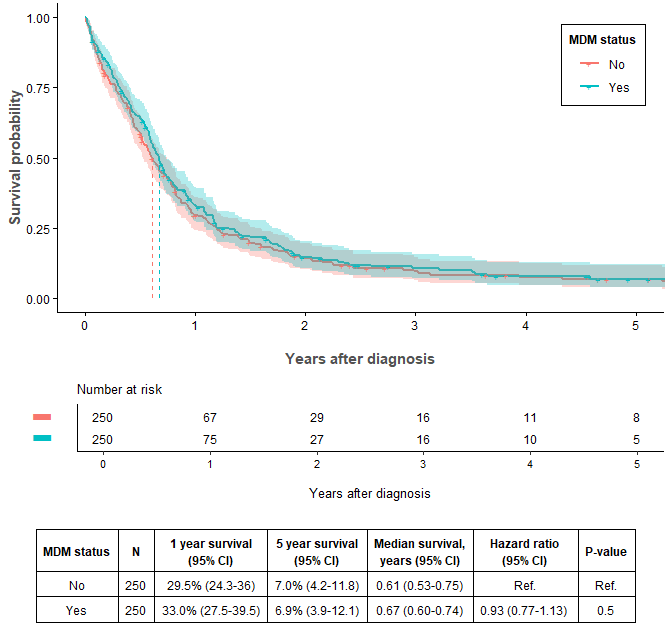

Supplement: Supplementary Figure 2 [file mmc6.docx]
